# Spatialities of Dog Theft: A Critical Perspective

**DOI:** 10.3390/ani9050209

**Published:** 2019-04-30

**Authors:** Daniel Allen, Adam Peacock, Jamie Arathoon

**Affiliations:** 1 School of Geography, Geology and The Environment, Keele University, Keele ST5 5BG, UK; a.j.peacock@keele.ac.uk; 2 School of Geographical and Earth Sciences, University of Glasgow, Glasgow G12 8QQ, UK; j.arathoon.1@research.gla.ac.uk

**Keywords:** dog theft, pet theft, dogs, pets crime, animal geography, GIS

## Abstract

**Simple Summary:**

Dogs are considered property under U.K. law, while owners generally regard their canine companions as family. Reports that the number of stolen dogs in England and Wales rose from 1788 in 2016 to 1909 in 2017 led to public calls to change the law. Recognising that a more robust analysis of dog theft crime statistics is required, we gathered dog theft data for 2015, 2016, and 2017 from 41 of 44 police forces. This paper examines how dog theft crime statistics are constructed, assesses the strengths and weaknesses of these data, and categorises, maps, and measures dog theft changes temporally per police force in England and Wales. Our findings reveal there has been an increase in dog theft crimes, with 1559 thefts in 2015, 1653 in 2016 (+6.03%), and 1842 in 2017 (+11.43%), and a decrease in court charges related to dog theft crimes, with 64 (3.97%) in 2015, 51 (3.08%) in 2016, and 39 (2.11%) in 2017. The actual number of dog theft crimes will be higher as three forces could not supply useable data. There is a need for a qualitative study to understand dog theft crime in different parts of the country, and a standardised approach to recording dog theft by all police forces in England and Wales. We recommend classifying dog theft (or pet theft more generally) as a crime in itself under the Sentencing Guidelines associated with the Theft Act 1968.

**Abstract:**

Dogs are considered property under U.K. law, while current discourses of pet ownership place canine companions as part of an extended family. This means sentences for those who steal dogs are not reflective of a dogs’ sentience and agency, rather in line with charges for those who steal a laptop or wallet. This is particularly problematic as dog theft is currently on the rise in England and Wales, leading to public calls to change the law. Recognising that a more robust analysis of dog theft crime statistics is required, we gathered dog theft data for 2015, 2016, and 2017 from 41 of 44 police forces through Freedom of Information (FOI) requests. This paper uses these data to examine how dog theft crime statistics are constructed, assesses the strengths and weaknesses of these data, and categorises, maps, and measures dog theft changes temporally per police force in England and Wales. Our findings reveal there has been an increase in dog theft crimes, with 1559 in 2015, 1653 in 2016 (+6.03%), and 1842 in 2017 (+11.43%), and a decrease in court charges related to dog theft crimes, with 64 (3.97%) in 2015, 51 (3.08%) in 2016, and 39 (2.11%) in 2017. There were police force inconsistencies in recording dog theft crime, which meant some data were unusable or could not be accessed or analysed. We recommend a qualitative study to understand stakeholder perspectives of dog theft crime in different areas, and a standardised and transparent approach to recording the theft of a dog by all forces across England and Wales. This could be achieved by classifying dog theft (or pet theft more generally) as a crime in itself under the Sentencing Guidelines associated with the Theft Act 1968.

## 1. Introduction

Under U.K. law, pets including dogs are regarded as “property” and pet theft is not classified as a specific crime in itself. Sentencing within the Theft Act 1968 is dependent on the monetary value of the stolen animal (under or above £500), and the crime is treated as a category three (fine to two years in custody) or category four offence (fine to 36 weeks in custody) in magistrates’ court [1,2]. Despite this legal status, in social terms dogs are generally recognised as members of the family [3,4,5]. They are loved, cared for, and accepted as individuals with unique personalities and emotional significance [6]. As such, a significant tension between the social and political dimensions of pet ownership continues to be exacerbated, whereby the sensual experiences of pet engagement are poorly represented through the legalities of U.K. law. Such tension has influenced respective campaigns by the Stolen and Missing Pets Alliance (Sampa) [7] and Dogs Trust [8] to reform the Theft Act 1968 [1] and its associated Sentencing Guidelines [2].

Scholars of human–animal studies maintain that dogs actively shape relations between family members who change everyday practices to incorporate the needs of their dogs [9,10,11]. The rise in positive training, for example, equates to the recognition of sentience and mindedness of dogs [12], and the emotional, affective, and caring relationships they facilitate. Fox argues further that in shaping everyday familial practices animal companions are intrinsic to our “sense” of home and belonging [3]. Practices such as grooming, walking, and playing not only show the embedding of “doggy-ness” into family life but are also practices of human care for their animals [13]. Human care can similarly be expressed through a greater diversity and commercialization of pet-related commodities including “doggy spas” and groomers, pet boutiques, pet hotels, high-end nutritional food markets, pet cemeteries, and even pet activities such as “dog yoga” or ”doggy dancing” [3,14,15]. While some of these commodities seem eccentric, they signify an emerging culture of care and the importance of pets to human lives in contemporary society as animals of love and affection. Furthermore, dogs also care for humans through actively embedding physical activity into their lives, providing security and safety, emotional support, and the ability to navigate safely [16,17,18]. Other studies point to how dogs also facilitate social interaction by acting as a social stimulus through making people more approachable, being a subject for idle chat of shared interests and helping provide a sense of community [19,20]. 

The human–dog relationship is characterised by affectionate caring practices that work relationally; both human and dog share a unique emotional bond with one another. Self-identification as “pet parents” has become a dominant discourse in western society, as has regarding dogs as “furry children” [4,5,10]. Ascribing names, feelings, and personalities may be forms of anthropomorphism, but it is a process that allows humans to relate to animals [3]. Experienced and embodied through trust, bodily gestures, and emotional investment, the ability to affect and be affected extends beyond and between human and animal bodies [21]. This can be expressed through owner’s recognition of their pets as minded individuals capable of empathizing and comforting them―a form of mutual communication [22]. Haraway maintains that humans and dogs are bonded by “significant otherness”, in “varied webs of interspecies dependence” [23,24]. Significantly, many people talk about the emotional difficulties of a pet’s death and how it is comparable to the loss of a loved one within the family [15,25]. Framing pets as both irreplaceable and grievable [14,25] in this way shows a deeply embodied and emotional relationship of care and companionship [6]. 

**Figure 1:**
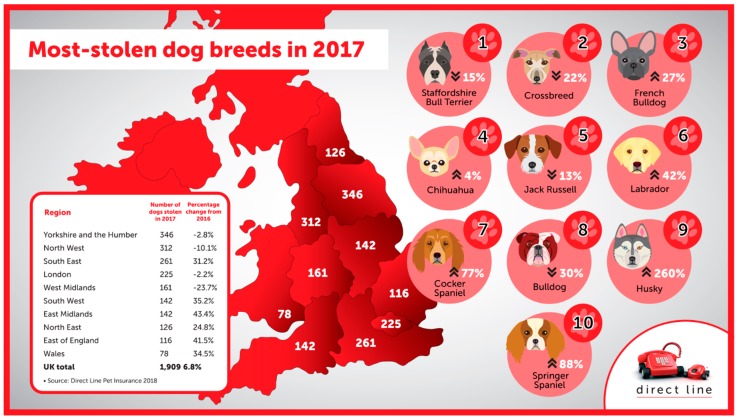
Most stolen dog breeds and total number of dogs stolen by regions of England and Wales in 2017 and associated changes since 2016 [27].

Dog theft is a crime that exploits these relationships [26], and is on the rise in the United Kingdom. Freedom of Information (FOI) research conducted by Direct Line Insurance revealed that the number of dogs reported stolen in England and Wales rose from 1788 in 2016 to 1909 in 2017 [27]. As can be seen in Figure 1, these data are useful for identifying the “most-stolen dog breeds”, information which can inform dog insurance policies and help raise public awareness through media campaigns. 

A similar study by Emporium Insurance showed 1712 thefts in 2015, 1803 in 2016 and 1977 in 2017 [28]. Media headlines included: “Lincolnshire dog theft capital of Britain” [29] and “Dog thefts increase by more than 200% in Dyfed-Powys area in just one year” [30]. Public support for pet theft reform grew in 2018. A petition to “Reclassify the theft of a pet to a specific crime in its own right” gained 107,353 signatures in six months and was debated in Parliament on 2 July 2018 [31]. Despite cross-party support, George Eustice MP concluded that “at the moment the Government are not convinced that we need to change the law”, but stressed “that the Government interpret the latest guidance from the Sentencing Council that the theft of a pet should generally be treated as a category two or three offence”. Alongside this, Eustice acknowledged “the need for statistics” [32]. The following day, Ross Thomson MP presented the first reading of the Pets (Theft) Bill in the House of Commons; its aim was “to amend the Animal Welfare Act 2006 and the Animal Health and Welfare (Scotland) Act 2006 to make the theft of pets an offence” [33].

Recognising that a more robust analysis of dog theft crime statistics is required, this paper (1) examines how dog theft statistics are constructed; (2) assesses the strengths and weaknesses of these data; and (3) categorises, maps and measures dog theft changes temporally per police force in England and Wales for 2015, 2016, and 2017. 

## 2. Materials and Methods

The analyses presented within this paper are the product of a two-step process. The former involved a data search for any information on dog theft across England and Wales, both qualitative and, predominantly, quantitative sources of information. Objectively, this allowed key objectives to be answered: (1) How are dog theft statistics being constructed? and (2) What are the strengths and weaknesses of these data? The second step involved attempting to collect these data ourselves from the 44 police precincts across England and Wales, via the Freedom of Information Act, in order gain a spatial representation of the prevalence of these crimes across the date range 2015–2017. This was completed using ArcGIS software (Esri, Collin TX, USA). Maps included in this paper were created using ArcGIS^®^ software by Esri. ArcGIS^®^ and ArcMapTM are the intellectual property of Esri and are used herein under licence. Copyright^©^ Esri. All rights reserved. For more information about Esri^®^ software, please visit www.esri.com. The process of each of these steps is outlined below:

### 2.1. Data Search for Dog Theft Data

Despite the increasing prevalence of dog theft across England and Wales [26,27], our own research demonstrated that data pertaining to such thefts are either particularly coarse/vague and missing large chunks of information―or altogether lacking. An initial data search brought two key sources of dog theft datum to our attention: one compiled by Direct Line Insurance and one compiled by Emporium Insurance. Both data sets were explored in order to try and deconstruct how dog theft statistics are calculated on the national scale and, indeed, to explore the relative strengths and limitations of each data set.

The data supplied by Direct Line and Emporium had followed similar methods of data collection to our own. Via the Freedom of Information Act the company had sent requests to each of the 44 police forces across the England and Wales. In the case of Direct Line, 41/44 forces responded to their requests for the years 2016–2017. The data provide a useful picture of the number of dogs stolen by region and further provided a list of the top ten breeds of dogs which are stolen. However, there were a number of issues. Firstly, the information sheet which is attached to these data groups the data on a regional basis, despite the geography being provided via each force. Similarly, no graphical or cartographical information is provided. More problematically, however, the data focus on the number of dogs stolen and not the number of crimes, failing to provide a breakdown as to the crime rate for each force. The number of dogs taken cannot be representative of the crime level as any number of dogs could be taken at one time, particularly given the increase in crime targeting dog breeders where multiple dogs can be taken during one burglary.

Comparatively, the data provided by Emporium Insurance included 38/44 police forces across England and Wales for number of dogs stolen. Only 26/44 police forces provided data for number of crimes, with three of those 26 sources being particularly coarse. There was greater detail in the different breeds which had been taken and the data did provide a figure as to the number of crimes that had occurred, as well as the number of dogs that were taken. This again illustrates the above point regarding using the number of dogs stolen as an indicator; their data show that, for example, in 2015 in the West Yorkshire police force jurisdiction, 184 dogs were stolen, but only 164 crimes were recorded as having occurred, misrepresenting the crime rate. 

Thus, such an analysis of existing data reveals a three-fold problem with the ways in which national statistics related to dog theft are being calculated. On the one hand, there is data for some police forces missing entirely, meaning the statistics are not fully representative of the dog theft situation across England and Wales. Of more prominent concern, however, is significant discrepancy between the number of forces’ data collected. Given each company devises their own statistics based on their respective data, then it becomes increasingly problematic to track a true, definitive, national picture of this issue. Finally, the use of the number of dogs stolen as a representation of the amount of crime is also problematic, as using data of this type neglects to consider the quantities of dogs that could be taken. The number of dogs is not equivalent to the number of crimes being committed. Given we have used data from two key insurance companies, if other companies are generating similar statistics with varying levels of information, then there is a clear need to group these data together in order to develop a more detailed, singular and national focus on dog theft. Indeed, this comprises the focus of stage two of the methods.

### 2.2. Data Collection and Geographic Information System (GIS) Analyses

In order to begin to further deconstruct the notion of “pet theft”―its respective characteristics and spatial prominence within England and Wales―we sent our own FOI requests to each of the 44 police forces across England and Wales. We asked two distinctive questions in relation to dog crime:

What was the total number of dog theft crimes in 2015/2016/2017?

What was the outcome (charge/summons, community resolution, active investigation, evidential difficulties, no suspect identified) for each dog theft crime in 2015/2016/2017?

We received 41/44 responses for the police forces across England and Wales. Some of the data from different forces were more detailed than others, whereby some forces would provide just a yearly figure as to how many thefts the system had returned upon using the key word “dog” in the forces’ system search, whilst others provided a breakdown of, for instance, where the crimes had been committed (i.e., in a residential building, in a car, or in a public setting). However, this level of detail was seldom given by many police forces. Hampshire, Sussex, and Wiltshire police forces were unable to provide any data or did not reply to requests under freedom of information legislation. Initially, Humberside Police force was able to provide some information; however, they admitted that these data were limited as the search had been stopped due to the cost/time it would take for these forces to gather the data:

"Although excess cost removes the forces obligations under the Freedom of Information Act, as a gesture of goodwill, I have supplied information, relative to your request, retrieved or available before it was realised that the fees limit would be exceeded. I trust this is helpful, but it does not affect our legal right to rely on the fees regulations for the remainder of your request."

A later request, however, led to a more detailed response. Thus, we were able to compile data for 41/44 forces for dog theft crime.

The data from each force were compiled in to one larger database―a lengthy process given the different ways the data had been presented to us. This process entailed sorting the data in to categories, through which some categories, those in Table 1, were created and represent an amalgamation of different responses (individually described below). We deemed this exercise important because, as previously described, the data had some discrepancies. For example, the data we were given were entered under the sub-heading “total claims of dog related crime” for each of the three years. However, this was not taken as the absolute value as there were instances where a “dog crime” was provided, but was not technically a theft. For instance, a lack of detail in data from West Yorkshire in 2017 prevents breaking the data down and therefore the assumption is that these were all thefts, whereas data from the West Midlands in 2017 indicate one recording of dog-related crime was fear/ provocation of violence, but this is not technically theft and therefore is not included in the total thefts category. Thus, the data were sorted as best as possible in order to “clean” them. We do, however, acknowledge that despite our best efforts the data are by no means perfect. 

### 2.3. GIS Spatial Mapping

Once the data had been managed we then categorised them using seven classes chosen due to the best number for representing colour categories (Table 2 below). This measured the number of reported crimes in quantities of 39. Maintaining these classes enabled comparisons of how the number of dog thefts has risen or fallen for each police force over the years of interest to the study. The objective here was not to compare the data between forces, as despite sorting the data there were unavoidable biases relating to how the data had been managed or input in to the system (owing to the lack of treating pet theft as a crime in its own right), but to show the spatial variation registered dog thefts by different forces. 

Once classes had been assigned, the database was imported in to ArcMap and joined with the Police Force Areas (December 2016) Shapefile provided by the Office for National Statistics [34]. The same colour symbology was adopted consistently for each of the categories across each of the years.

### 2.4. Crime Rates

For crime rates to be calculated, the estimated population of police force areas were identified from the Office for National Statistics for 2015, 2016, and 2017. The annual number of dog theft crimes per force were divided by the estimated population then multiplied by 100,000. 

## 3. Results and Analysis

### 3.1. Theft in England and Wales 

Dog theft crime is not recognised by the Office for National Statistics on their “Crime in England and Wales: Police force area data tables”. Therefore, FOI requests are the only way to access these data. Table 3 shows the various classifications of theft used by Police Forces and the Sentencing Council—these figures are available in the public domain through the Office of National Statistics [35]. Police forces can label dog theft and pet theft as burglary, domestic burglary, vehicle offences, theft from the person, and other theft offences. The number of thefts rose annually from 1,762,473 in 2015 to 1,820,079 in 2016 (up 3.26%) and 2,011,942 in 2017 (up 10.54%) as shown in Table 3. It is important to situate our research with broader theft offences within the United Kingdom. 

### 3.2. Dog Theft in England and Wales

**Figure 2:**
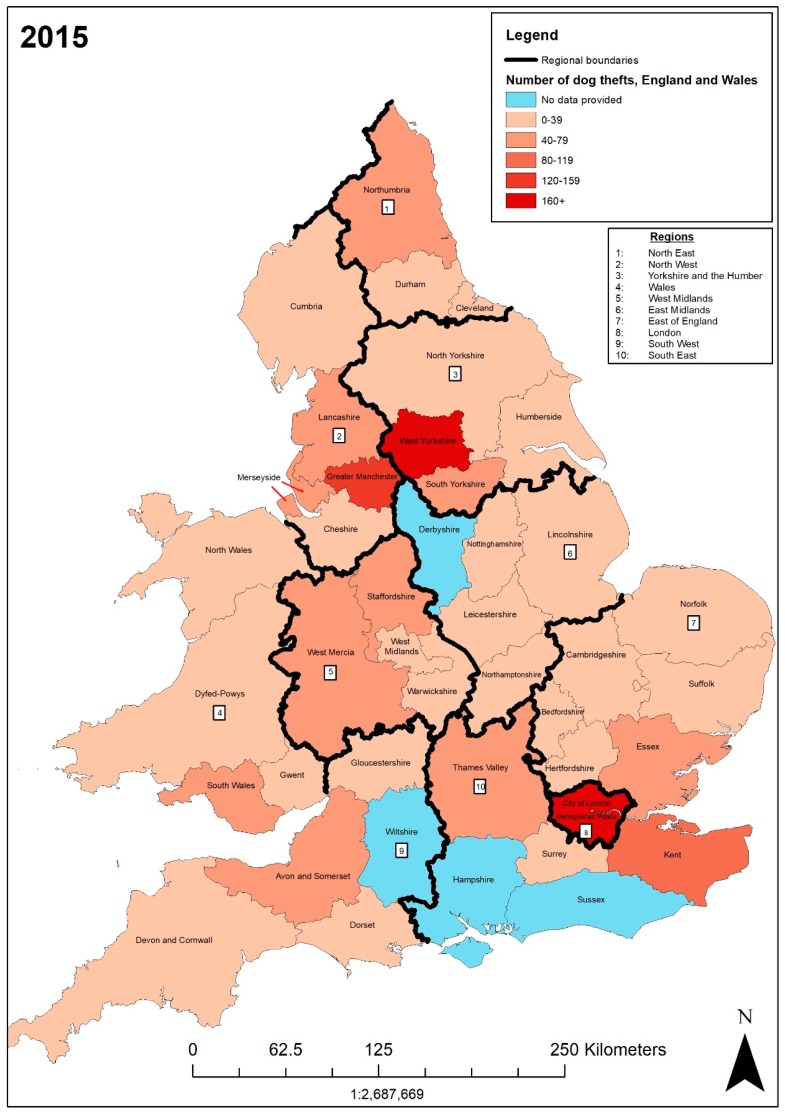
Number of dog theft crimes recorded by each police force in England and Wales in 2015.

In 2015, the forces with the highest numbers of dog theft crimes (DTCs) were the Metropolitan police (167) and the West Yorkshire (166), Greater Manchester (120), Kent (102), and Essex (74) police. The lowest number of recorded dog theft crimes was in Cheshire (6). This is indicated on the map in Figure 2. The dark red colour specifies 160+ DTCs (i.e., Metropolitan and West Yorkshire police), whereas the pale orange specifies 0–39 DTCs (i.e., Cheshire police). The City of London had no recorded dog theft crimes―this was not mapped as the force only covers a 2.8 km² area. The blue shows the police force areas in which we had no data (i.e. Wiltshire police). Overall, 25 police forces recorded between 0 and 39 dog theft crimes. Furthermore, Figure 2 shows that some neighbouring police forces of the Metropolitan area, West Yorkshire, and Greater Manchester have 40–79 or 80–119 DTCs, for example, Thames Valley (47), Essex (74), Kent (102), South Yorkshire (58), Merseyside (53), and Lancashire (59), Category 40–79 also includes West Mercia (43), Northumbria (44), Avon and Somerset (48), Staffordshire (55), and South Wales (62). The three areas with the greatest number of DTCs—the Metropolitan area, West Yorkshire, and Greater Manchester—also had some of the highest numbers of police per 100,000 people, rating first, tenth, and fifth, respectively, and hold greater populations—as shown on Table 4. 

The rates of dog theft crime charges also varied. In Greater Manchester there were six charges from 120 dog theft crimes (5%) whereas in Northumbria there were 44 dog theft crimes with six charges (13.64%). The FOI requests do not, however, make it clear whether charges for dog theft crime relate to multiple dog thefts or one. This is important as this can misconstrue the crime rate. FOI requests were made to the Ministry of Justice to access information on sentences—their response stated “centrally held information cannot identify dog theft from other theft. Therefore any request for this information is likely to require a manual search of all sentenced cases related to theft”. With “over 77,000 sentences handed down by courts in England and Wales cases related to theft”, accessing these data is near impossible [36].

Exploring DTCs per 100,000 people (DTC rates), we found that West Yorkshire ranked highest (7.27), followed by Kent (5.66), Gwent (5.15), Staffordshire (4.93), South Wales (4.74), and Greater Manchester (4.35). The lowest rates of DTCs per 100,000 were in Cheshire (0.57) and City of London (0). The Metropolitan police (1.92) had a relatively low rate of DTCs per 100,000 compared to the number of dog theft crimes (167). While the Metropolitan police had highest DTC crimes in 2015 and currently the most police officers per 100,000, the DTC rate per 100,000 was low due to the Metropolitan area having the greatest population in England and Wales. On the other hand, Staffordshire (4.93) had a relatively high rate of DTCs per 100,000 compared to the number of dog theft crimes (55). They are ranked 41st for police per 100,000 people. Overall, the police force data available to us through FOI requests showed 1559 dog thefts nationally in 2015. The outcome of 853 cases was “no further action” (54.71%), and there were 64 charges (3.97%). 

**Figure 3:**
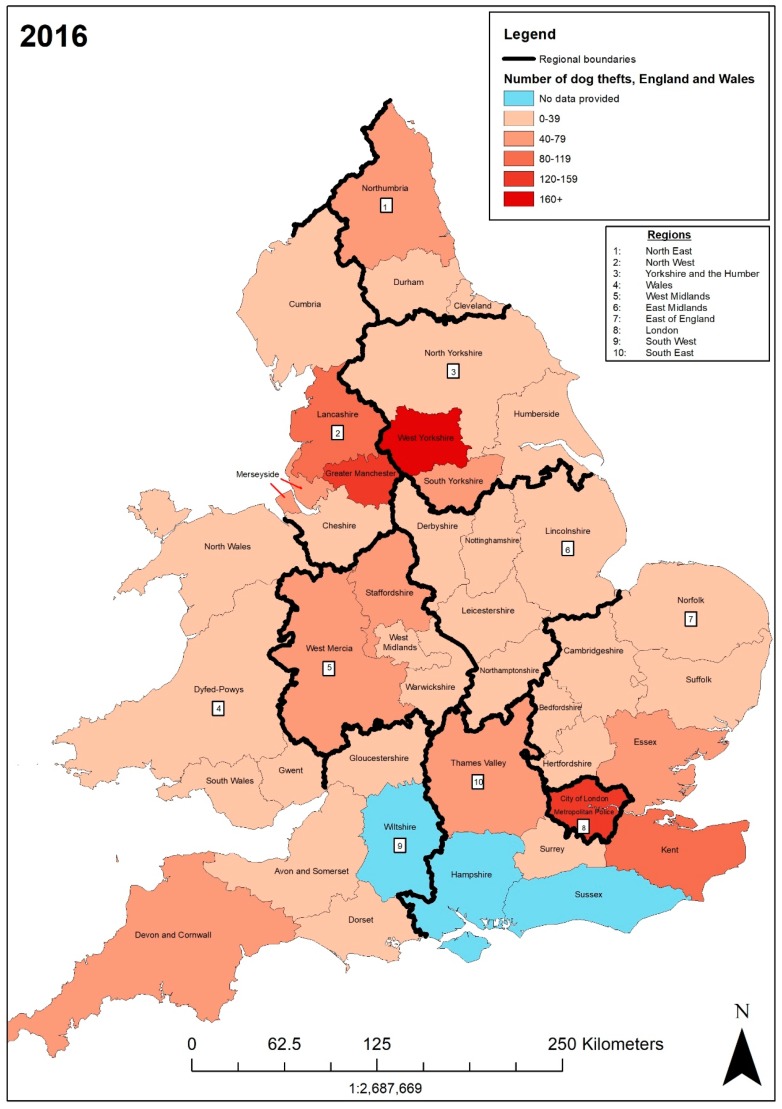
Number of dog theft crimes recorded by each police force in England and Wales in 2016.

In 2016, the forces with the highest numbers of dog theft crimes were West Yorkshire (197), Metropolitan (137), Greater Manchester (132), Kent (107), and Lancashire (100). The lowest recorded numbers of dog theft crimes were in Surrey (10) and City of London (0). In Figure 3, West Yorkshire is shown in the category 160+, the Metropolitan area and Greater Manchester in the category 120–159, and Kent and Lancashire in the category 80–119. The four police forces experiencing the highest number of DTCs in 2015 are also experiencing the highest in 2016. There is some movement with West Yorkshire recording 197 DTCs (+31) and the Metropolitan recording 137 (−30). Furthermore, Lancashire replaces Essex, with the fifth highest DTC rate. Surrey has the lowest number of DTCs with 10, whereas Cheshire―the lowest in 2015 with 6―increased by 13. Surrey and Cheshire remain in the 0–39 category, which has 23 police forces. Additionally, a pattern recognised for the mapped 2015 data―neighbouring policing areas of Metropolitan, West Yorkshire, and Greater Manchester police forces had higher rates of DTCs, decreasing outwards―is also shown in Figure 3.

The forces with an annual increase in DTCs from 2015 to 2016 include Lancashire with 100 (+41), West Yorkshire with 197 (+31), Devon and Cornwall with 62 (+26), West Mercia with 61 (+18), Northumbria with 61 (+17), Staffordshire with 68 (+13), Cheshire with 19 (+13), Great Manchester with 132 (+12), Merseyside with 65 (+12) Durham with 23 (+10) Cambridgeshire with 25 (+9), Lincolnshire with 21 (+7), South Yorkshire with 65 (+7), Cleveland with 22 (+6), Dorset with 12 (+5), Kent with 107 (+5), North Wales with 21 (+5), Derbyshire with 4 (+4, data unavailable in 2015), North Yorkshire with 23 (+2), Gwent with 30 (+1), and Nottinghamshire with 16 (+1). The forces with an annual decrease in DTCs include South Wales with 27 (–35), the Metropolitan area with 137 (−30), Avon and Somerset with 21 (–27), Norfolk with 15 (–17), Northamptonshire with 11 (−14), Essex with 67 (−7), Suffolk with 12 (−6), Gloucestershire with 13 (–5), Hertfordshire with 18 (−5), Dyfed-Powys with 14 (−4), Cumbria with 12 (−3), Humberside with 31 (−2), and Surrey with 10 (−2). Overall, there was a yearly increase in DTCs of 6.03% from 2015 to 2016.

In Northumbria there were seven charges from 61 DTCs (11.48%), compared to Greater Manchester with three charges from 132 DTCs (2.27%). Again a similar issue arises as FOI requests do not, however, make it clear whether charges for dog theft crime relate to multiple dog thefts or one. This is important as this can misconstrue the crime rate.

In 2016 West Yorkshire retained the highest-ranking DTC rate per 100,000 (8.58), followed by Lancashire (6.73), Staffordshire (6.07), Kent (5.88), and Gwent (5.30). The lowest rate of DTCs per 100,000 was in Surrey (0.84). West Yorkshire had the most DTCs in 2016 and also the highest DTCs per 100,000 people, whereas Surrey had both the lowest DTC number in 2017 and the lowest DTCs per 100,000 people. Staffordshire’s DTC rate per 100,000 increased from 2015 (4.93) to 6.07, as it recorded 13 more DTCs in 2016. The Metropolitan police still have a relatively low DTCs per 100,000 (1.56) compared to its second highest number of DTCs of 137 (167 DTCs, 1.97 crimes per 100,000 in 2015). Overall, there were 1653 dog theft crimes in 2016, an annual increase of 6.03%. Of these, the outcome of 1013 ended with no further action (61.28%―a 6.57% increase from 2015), and 51 charges (3.08%).

**Figure 4:**
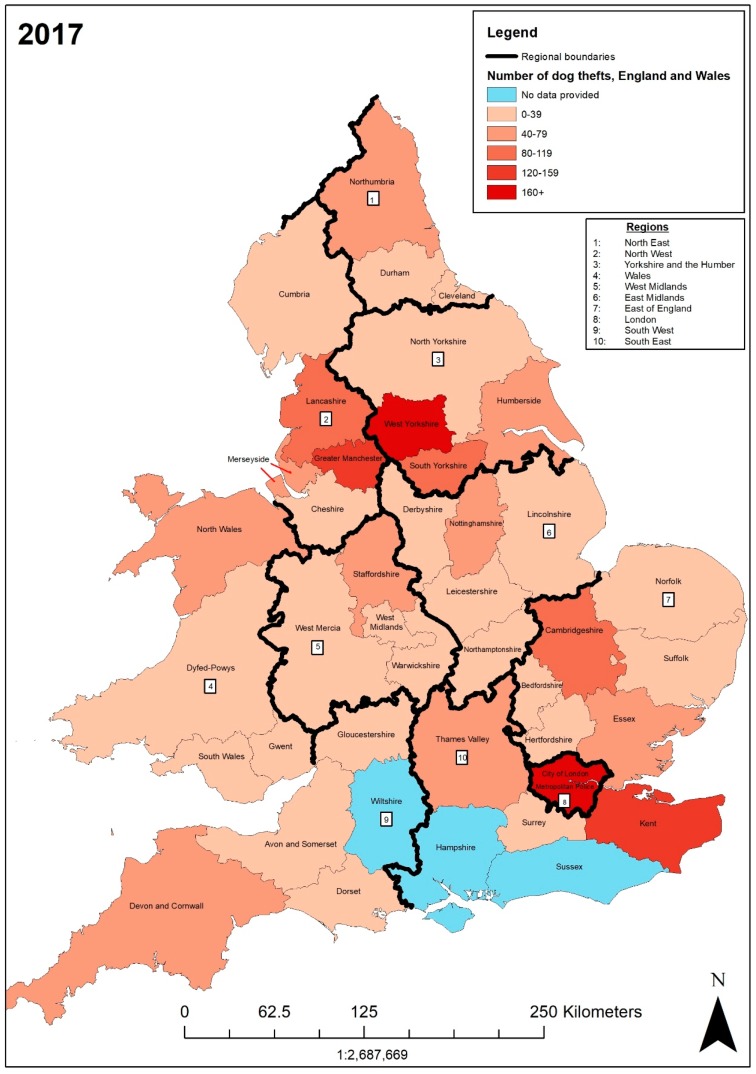
Number of dog theft crimes recorded by each police force in England and Wales in 2017.

In 2017, the forces with the highest numbers of dog theft crimes were West Yorkshire (172), Metropolitan (169), Greater Manchester (146), Kent (130), and Lancashire (93). The lowest recorded dog theft crime numbers were in Cheshire (4) and the City of London (0). Similarities emerge from the 2016 data as the five police forces with the highest DTCs in 2016 also had the greatest number of DTCs in 2017. After having an increase of 13 DTCs from 2015–2016, Cheshire then recorded the lowest number of DTCs in 2017. In Figure 4 the West Yorkshire and Metropolitan police forces are in the 160+ category, Greater Manchester and Kent in the 120–159 category, and Lancashire in the 80–119 category. Cheshire is in the lowest category 0–39 with 23 other police forces. Additionally, a pattern continues for the mapped 2015 and 2016 data―the neighbouring policing areas of Metropolitan, West Yorkshire, and Greater Manchester police forces have higher DTCs, decreasing outwards. This is also shown in Figure 3.

The police forces which reported increasing dog theft crimes were Metropolitan with 169 (+32), Nottinghamshire with 43 (+27), Thames Valley with 72 (+25), Kent with 130 (+23), North Wales with 40 (+19), Devon and Cornwall with 78 (+18), Dyfed-Powys with 31 (+17), South Yorkshire with 82 (+17), Cambridgeshire with 41 (+16), Durham with 37 (+14), Greater Manchester with 146 (+14), Norfolk with 29 (+14), Northumbria with 74 (+13), Humberside with 44 (+13), Cumbria with 24 (+12), Derbyshire with 11 (+7), Bedfordshire with 20 (+4), Dorset with 16 (+4), Northamptonshire with 15 (+4), West Midlands with 29 (+4), Cleveland with 25 (+3), Lincolnshire with 24 (+3), Merseyside with 68 (+3), Staffordshire with 70 (+2), and Suffolk with 13 (+1). The police forces with decreasing rates of dog theft crime were West Mercia with 35 (−26), West Yorkshire with 172 (−25), Cheshire with 4 (−15), Essex with 52 (−15), South Wales 17 (−10), North Yorkshire with 15 (−8), Lancashire with 93 (−7), Leicestershire with 23 (−6), Surrey with 8 (−2), and Hertfordshire with 17 (−1). Gwent (31) and the City of London (0) had no change. Overall, from 2016 to 2017 there was a 11.43% increase in DTCs.

The numbers of people charged also varied. While Kent recorded 130 dog theft crimes in 2017, there were only four charges (3.07%). In Bedfordshire there were 20 dog theft crimes, with an outcome of four charges (20%). There is a vast difference between the number of dog theft crimes and the number of people charged for these Kent and Bedfordshire police forces. However, there is little difference between the number of police officers per 100,000 people (17th and 22nd, respectively) – as shown on Table 4.

The highest dog theft crime rates in 2017 per 100,000 were West Yorkshire (7.45), Kent (6.72), Lancashire (6.23), Staffordshire (6.21), and Dyfed-Powys (5.99). The lowest recorded dog theft crime rates per 100,000 people were in Cheshire (0.37) and the City of London (0). West Yorkshire had the greatest number of DTCs in 2017 as well as previously in 2016, and also had the highest DTC rate per 100,000 people from 2015 to 2017. Aside from City of London, which had an estimated population of 7700 and no recorded DTCs, Cheshire had both the lowest DTC in 2017 and the lowest DTCs per 100,000 people. Staffordshire’s DTC rate per 100,000 increased from 2016 (6.07) to 6.21 as it recorded two more DTCs in 2017 (70). The Metropolitan police still has a relatively low DTC per 100,000 (1.91) compared to its second highest number of DTCs of 169. Overall, there were 1842 dog thefts nationally in 2017, an annual increase of 11.43%. Of these, the outcome of 1013 was no further action (61.28%) and there were 39 charges (2.11%). Whilst there was an increase in dog theft crimes annually, there was also a decrease in the number of charges annually. 

Our findings, in Table 5, reveal there has been an increase in dog theft crimes in England and Wales, with 1559 in 2015, 1653 in 2016, and 1842 in 2017, and a decrease in court charges related to dog theft crimes, with 64 (3.97%) in 2015, 51 (3.08%) in 2016, and 39 (2.11%) in 2017. In each of those years the outcome of no further action remained relatively stable at 54.71% (2015), 61.28% (2016), 58.36% (2017). In the context of overall number of theft offences in England and Wales, there was a 3.26% increase in 2016 compared to a 6.03% increase in dog theft crimes, and 10.84% in 2017, in line with a 11.43% increase in dog theft crimes in 2017. These figures, however, are only indicative as three forces could not supply dog theft data. 

Our research also revealed wider discrepancies with the data provided from FOI requests by Emporium and Direct Line. Emporium, for example, stated 152 dogs had been stolen from Lincolnshire in 2017, compared to the 27 stolen dogs stated by Direct Line. Our own FOI data, which were the last to be requested, revealed only 24 dog theft crimes (Table 6). While we acknowledge these are “live” systems and “recording these figures are not generic, nor are the procedures used locally in capturing the crime data”, it makes little sense if “this force’s response to your questions should not be used for comparison purposes with any other response you may receive” [38].

Data from Merseyside also showed that FOI requests from both Emporium and Direct Line listed 29 stolen dogs in 2017, yet our FOI reveals 68 dog theft crimes. This suggests the number of stolen dogs supplied by Merseyside Police is incorrect or incomplete. Furthermore, this discrepancy in the numbers could be due to a lack of a universal recording system for dog theft crimes, as we have stated dog theft crimes can be recorded as anything from a domestic burglary to theft from a person.

## 4. Conclusions

In this paper we have examined how dog theft crime statistics are constructed, assessed the strengths and weaknesses of these data, and categorised, mapped, and measured dog theft changes temporally per police force in England and Wales. Our findings revealed an annual increase in dog theft crimes, from 1559 in 2015 to 1653 in 2016 (+6.03%) and 1842 in 2017 (+11.43%). Despite the year-on-year rise of dog theft crimes, court charges related to dog theft decreased from 64 (3.97%) in 2015 to 51 (3.08%) in 2016 and 39 (2.11%) in 2017. The proportion of court charges are significantly small in relation to crimes recorded.

We found both strengths and weaknesses within the data we used. Firstly, although our dog theft crime database provides the most robust dog theft crime data available for England and Wales in 2015, 2016 and 2017, the dataset remains incomplete. There were police force inconsistencies in recording dog theft crime, which meant some data was unusable or could not be accessed or analysed. This has implications on the accuracy of any spatial analysis. Secondly, FOI requests are the only way to access this data. This led to some of the data from some police forces not being provided due to the cost and time requirements of searching and providing the data. As a result, three of 44 police forces could not supply useable data. 

Other issues arose due to dog theft not being classified as a crime in its own right. This showed through a lack of universal recording and the data being very vague or coarse. The quantitative analysis of police force data we have presented only provides a superficial understanding of dog theft crime in the areas included—the figures do not reveal why dog theft crime is increasing or decreasing. Furthermore, our approach has not taken into consideration the experiences of those involved in dog theft. Pet ownership is a highly emotional, affective, and caring practice, and pets are important in shaping the lives of humans [3,6,13]; stakeholder perspectives would provide more detailed insights. 

Consequently, we have two key recommendations. First, there is a need for a qualitative study to understand dog theft crime in different parts of the country. While our approach was important, it did not tell us why dog theft crime and related charges increased or decreased annually from 2015 to 2017. A qualitative study with victims of dog theft, theft support organizations, police officers, and convicted dog thieves would help understand why dog theft crime increased/decreased in certain areas. Furthermore, as we have shown, some police forces have a higher percentage rate of people charged for dog theft crimes, and others have seen a decrease in dog theft crimes. A qualitative study is required to understand stakeholder perspectives of dog theft crime in specific areas. This would take into account police force strategies, external factors such as the media engagement and organizational collaboration, and the experiences of victims of dog theft crime. Alongside this, the databases of stolen and missing pet organisations could also be analysed to gain a better quantitative understanding of the spatialities of dog theft.

Second, there is a need for a standardised approach to recording dog theft to help provide greater transparency with respect to dog theft crimes in England and Wales. This could be achieved by classifying dog theft (or pet theft more generally) as a crime in itself under the Sentencing Guidelines associated with the Theft Act 1968. Classifying dog theft in this way would also help compare and contrast the spatiality of dog theft crime, providing details from police force jurisdictions with greater problems with dog theft crime. The classification of dog theft as a specific crime would also reflect the greater public discourse around dog ownership and help situate them as emotional sentient beings rather than disposable inanimate objects.

## Figures and Tables

**Table 1:** Any “merged” categories that were made during the data sorting process. These were rational decisions made to manage the data given the vast differences between how the data are recorded or how they were presented to us.

Merged Categories:	Explanation/Rationale:
Theft in public:	Any instance where the pet was taken in the public, such as at an ATM or in a store. This does not include thefts taking place in vehicles (separate classification, below) or thefts taking place within buildings deemed as businesses.
Theft of/from a vehicle:	Any instance where a pet was taken from a car, or was within a car when the vehicle was stolen.
Evidential difficulties:	This includes instances where either the police deemed there not to be enough evidence to proceed, thus closing the case, or where the public were willing to cooperate but could not provide evidence.
Withdrawn support/unwilling to assist:	Instances where the public could not or did not cooperate with the police investigation, therefore closing the case. This is separated from evidential difficulties as it could demonstrate false reporting, such as a domestic dispute over pet ownership etc.
Not in public interest (police decision)/prevented:	This includes instances where the crime was classified as “prevented”―the assumption is that the crime did not happen. It also includes instances where the police deemed the prosecution as not being worth pursuing, usually because the dispute was settled.
No further action:	Instances where “no further action” was listed in the data, where the case was cancelled or transferred by the force, or where the case was falsely recorded.
Penalty notice/caution/other:	Any instance where a penalty notice or fine was implemented as punishment, where a police caution was given instead of an arrest, or where a court summons is recorded but the outcome of which is not described further. This also includes categories such as youth restorative programmes, extended professional opinions, situations where complaints were made, or where another force had primacy jurisdiction. It also includes court disposal of cases or instances where prosecution time was marked as “expired”.

**Table 2:** Class ranges used across the data set for the purposes of spatial mapping.

Class	A	B	C	D	E	F
**Range**	Null	0–39	40–79	80–119	120–159	160+

**Table 3:** Crime in England and Wales (adapted by the authors from Office for National Statistics data).

Year	Theft Offences	Burglary	Domestic Burglary	Non-Domestic Burglary	Vehicle Offences	Theft from the Person	Bicycle Theft	Shoplifting	Other Theft Offences
2015	1,762,473	401,718	193,851	207,867	364,468	82,384	87,895	333,671	492,337
2016	1,820,079 (+3.26%)	404,282 (+0.63%)	200,659 (+3.51%)	203,623 (−2.04%)	389,371 (+6.83%)	86,548 (+5.05%)	90,910 (+3.43%)	358,235 (+7.36%)	490,733 (−0.32%)
2017	2,011,942 (+10.54%)	438,971 (+8.58%)	288,728 (+43.88%)	150,243 (−26.21%)	452,683 (+16.16%)	99,101 (+14.50%)	102,581 (+12.83%)	385,265 (+7.54%)	533,341 (+8.68%)

**Table 4:** FOI from 2015, 2016, and 2017 with associated crime rates per 100,000 people.

Police Force Area	Number of Dog Theft Crimes, 2015	Crime Rate Per 100,000, 2015	Number of Dog Theft Crimes 2016	Crime Rate Per 100,000 2016	Number of Dog Theft Crimes, 2017	Crime Rate Per 100,000, 2017	Police Per 100,000 Population (Rank) [37]
Avon and Somerset	48	2.88	21	1.24	21	1.23	153 (35)
Bedfordshire	15	2.29	16	2.41	20	3.00	170 (22)
Cambridgeshire	16	1.90	25	2.97	41	4.83	163 (32)
Cheshire	6	0.57	19	1.81	4	0.37	192 (15)
Cleveland	16	2.84	22	3.89	25	4.41	222 (6)
Cumbria	15	3.01	12	2.40	24	4.81	220 (8)
Derbyshire	No Data	No Data	4	0.38	11	1.04	166 (26)
Devon and Cornwall	36	2.09	62	3.57	78	4.45	169 (23)
Dorset	7	0.91	12	1.56	16	2.07	164 (31)
Durham	13	2.07	23	3.66	37	5.87	181 (16)
Dyfed-Powys	18	3.48	14	2.71	31	5.99	229 (3)
Essex	74	4.14	67	3.70	52	2.85	162 (33)
Gloucestershire	18	2.91	13	2.08	10	1.59	171 (20)
Greater Manchester	120	4.35	132	4.74	146	5.21	227 (5)
Gwent	30	5.15	31	5.30	31	5.27	216 (11)
Hampshire	No Data	No Data	No Data	No Data	No Data	No Data	143 (39)
Hertfordshire	23	1.97	18	1.53	17	1.43	165 (27)
Humberside	33	3.56	31	3.34	44	4.73	193 (14)
Kent	102	5.66	107	5.88	130	6.72	178 (17)
Lancashire	59	3.99	100	6.73	93	6.23	199 (13)
Leicestershire	24	2.27	29	2.71	23	2.12	164 (29)
Lincolnshire	14	1.90	21	2.81	24	3.19	145 (38)
Merseyside	53	3.79	65	4.60	68	4.79	244 (2)
Metropolitan Police	167	1.92	137	1.56	169	1.91	352 (1)
Norfolk	32	3.61	15	1.68	29	3.22	173 (19)
North Wales	16	2.30	21	3.02	40	5.74	214 (12)
North Yorkshire	21	2.59	23	2.81	15	1.82	164 (30)
Northamptonshire	25	3.45	11	1.50	15	2.02	219 (9)
Northumbria	44	3.06	61	4.22	74	5.10	165 (28)
Nottinghamshire	15	1.33	16	1.40	43	3.74	167 (25)
South Wales	62	4.74	27	2.04	17	1.28	220 (7)
South Yorkshire	58	4.21	65	4.69	82	5.88	176 (18)
Staffordshire	55	4.93	68	6.07	70	6.21	141 (41)
Suffolk	18	2.42	12	1.59	13	1.71	149 (37)
Surrey	12	1.02	10	0.84	8	0.67	168 (24)
Sussex	No Data	No Data	No Data	No Data	No Data	No Data	151 (36)
Thames Valley	47	1.99	47	1.97	72	3.01	170 (21)
Warwickshire	13	2.34	13	2.32	13	2.29	143 (40)
West Mercia	43	3.44	61	4.83	35	2.75	155 (34)
West Midlands	25	0.88	25	0.87	29	1.00	227 (4)
West Yorkshire	166	7.27	197	8.58	172	7.45	217 (10)
Wiltshire	No Data	No Data	No Data	No Data	No Data	No Data	139 (42)

**Table 5:** Number of dog thefts nationally and the number of outcomes in terms of no further action and charges.

Year	Number of Thefts Nationally	No Further Action (NFA)	% NFA	Number Charged	% Charged
2015	1559	853	54.71%	64	3.97%
2016	1653 (+6.03%)	1013 (+18.76%)	61.28%	51 (−20.31%)	3.08%
2017	1842 (+11.43%)	1075 (+6.12)	58.36%	39 (−23.53%)	2.11%

**Table 6:** Freedom of Information (FOI) data from Emporium, Direct Line and Allen et al.

Police Force Area	Number of Dogs Stolen 2017 (Emporium, FOI January 2018)	Number of Dogs Stolen 2017 (Direct Line, FOI February 2018)	Number of Dog Theft Crimes 2017 (Allen et al., FOI May 2018)
Avon and Somerset	19	21	21
Bedfordshire	17	18	20
Cambridgeshire	36	40	41
Cheshire	1	4	4
City of London	0	0	0
Cleveland	24	28	25
Cumbria	No Data	23	24
Derbyshire	11	11	11
Devon and Cornwall	80	80	65
Dorset	28	28	16
Durham	51	51	37
Dyfed-Powys	70	36	31
Essex	60	No Data	52
Gloucestershire	14	13	10
Greater Manchester	148	157	146
Gwent	14	12	21
Hampshire	No Data	No Data	No Data
Hertfordshire	22	17	17
Humberside	44	52	51
Kent	130	160	130
Lancashire	116	99	93
Leicestershire	23	27	23
Lincolnshire	152	27	24
Merseyside	29	29	68
Metropolitan Police	225	225	169
Norfolk	31	29	29
North Wales	No Data	No Data	40
North Yorkshire	15	19	15
Northamptonshire	15	34	15
Northumbria	47	47	74
Nottinghamshire	43	43	43
South Wales	22	30	17
South Yorkshire	108	103	82
Staffordshire	73	76	70
Suffolk	No Data	12	13
Surrey	9	8	8
Sussex	No Data	No Data	No Data
Thames Valley	No Data	93	72
Warwickshire	12	12	13
West Mercia	63	40	35
West Midlands	31	33	29
West Yorkshire	221	172	172
Wiltshire	17	No Data	No Data
